# Mediating role of peripheral thyroid hormone conversion efficiency in the association between BMI and IGF-1 in children with growth retardation: a cross-sectional study

**DOI:** 10.3389/fendo.2026.1941425

**Published:** 2026-09-17

**Authors:** Mengying Zhang, Hua Liu

**Affiliations:** Department of Child Health Care; Clinical Research Center of Child Health Care and Developmental & Behavioral Disorders, Institute of Maternal and Child Health, Wuhan Children’s Hospital (Wuhan Maternal and Child Health Care Hospital), Tongji Medical College, Huazhong University of Science & Technology, Wuhan, China

**Keywords:** body mass index, FT3/FT4 ratio, growth retardation, insulin-like growth factor-1, mediation analysis, prepubertal children

## Abstract

**Objective:**

To examine the association between body mass index (BMI) standard deviation score (SDS) and insulin-like growth factor-1 (IGF-1) SDS and whether thyroid hormones mediate it, focusing on the peripheral conversion marker free triiodothyronine/free thyroxine (FT3/FT4) ratio, in prepubertal children (2.0–9.0 years) with height SDS < –1.0.

**Methods:**

This retrospective cross-sectional study included 1,242 such children. Restricted cubic splines assessed the BMI-IGF-1 relationship, and Bootstrap mediation (5,000 resamples) tested indirect effects of FT3/FT4, FT3, FT4, and thyroid-stimulating hormone (TSH), adjusted for sex, age and height SDS. Reverse mediation, subgroup, interaction, Monte Carlo power, and E-value sensitivity analyses were also performed.

**Results:**

A significant linear positive association was found (P-overall < 0.001; P-nonlinear = 0.0719). FT3/FT4 and FT3 showed significant partial mediation, accounting for 9.12% (P < 0.001) and 11.60% (P = 0.0108) of the total effect, respectively; TSH and FT4 did not. Sensitivity analyses confirmed robustness after stricter prepubertal cutoffs and exclusion of overweight/obese children. Notably, only FT3/FT4 remained significant after excluding suspected Growth Hormone Deficiency (GHD) (IGF-1 SDS < –2.0). The FT3/FT4 ratio remained a significant mediator across all subgroup analyses. Reverse mediation analysis yielded a substantially smaller indirect effect in the reverse direction. E-values for FT3/FT4 and FT3 were 1.0764 and 1.0609, respectively.

**Conclusion:**

In this growth-retarded prepubertal cohort, BMI and IGF-1 are linearly associated. The FT3/FT4 ratio is statistically supported as a partial mediator with moderate effect size but strong statistical robustness, while the central TSH pathway is uninvolved, supporting a peripheral “thrifty” regulatory strategy and offering a potential target for nutritional intervention. These mediation findings reflect statistical associations and require prospective validation.

## 1 Introduction

Childhood growth retardation represents one of the most common chief complaints in pediatric endocrinology clinics. After excluding definitive etiologies such as growth hormone deficiency (GHD), hypothyroidism, chronic systemic diseases, and genetic syndromes, a considerable proportion of affected children are classified as having idiopathic short stature (ISS) or nutritional growth retardation (1–3). Clinically, many children presenting with growth concerns have heights between the 3rd and 16th percentiles for age and sex (height standard deviation score (SDS) between -2.0 and -1.0); although they do not meet the strict criteria for short stature, they already exhibit significant growth deceleration. For this subset, optimizing endogenous growth potential through modifiable external factors remains a pressing clinical question.

Nutritional status is a pivotal environmental regulator of the growth hormone-insulin-like growth factor-1 (GH-IGF-1) axis (4, 5). A recent large-scale general pediatric study (n = 3,227, aged 2–18 years) identified an inverted U-shaped curvilinear relationship between body mass index (BMI) and IGF-1, with a plateau or even decline observed at the upper BMI extreme (6). It should be noted, however, that this inverted U-shaped relationship was derived from a mixed population spanning puberty and obesity; the plateau or decline at the upper BMI range may be driven largely by pubertal sex steroid changes and obesity-related hepatic GH resistance. Whether a similar non-linear pattern exists specifically in prepubertal children with short stature and leanness remains largely unexplored. However, whether this association remains non-linear in a clinical population characterized predominantly by short stature and leanness, particularly during the strictly prepubertal period devoid of gonadal axis interference, remains unexplored. The answer to this question directly informs clinical nutritional intervention expectations — if a “ceiling effect” exists, excessive weight gain would be futile; if purely linear, even marginal improvements would confer sustained benefits.

The thyroid axis serves as a critical nexus linking energy metabolism and linear growth. Classic *in vitro* experiments by Schmid et al. have established that triiodothyronine (T3) directly stimulates IGF-1 synthesis (7). Conversely, IGF-1 has been shown to exert trophic effects on thyroid follicular cells, stimulating cell proliferation and modulating thyroid-specific gene expression, including thyroglobulin synthesis (8, 9). This bidirectional interplay between thyroid hormones and IGF-1 underscores the importance of considering potential reverse or reciprocal influences when interpreting the directional pathways in mediation models — the nutrition–thyroid–growth axis is not strictly unidirectional, and the possibility that IGF-1 may in turn modulate thyroid function and energy metabolism should be formally acknowledged. The free triiodothyronine/free thyroxine (FT3/FT4) ratio, an indicator of peripheral thyroid hormone conversion efficiency, reflects deiodinase activity — the tissue-level capacity to convert the prohormone thyroxine (T4) to the active T3 (10, 11). Related studies have found positive correlations of thyroid-stimulating hormone (TSH) and FT3 with BMI and a negative correlation of FT4 with BMI in obese children (12, 13); however, the regulatory network of the nutrition-thyroid-growth axis at the opposite end of the nutritional spectrum — in children with mild leanness/growth retardation — remains to be delineated. Crucially, no study to date has formally tested, within a mediation analysis framework, whether thyroid axis hormones, particularly the FT3/FT4 ratio, assume a statistical transmission role in the BMI–IGF-1 association.

Therefore, targeting prepubertal children aged 2–9 years presenting with short stature/growth retardation (height SDS < -1.0), this study aimed to address two core questions: (1) What is the shape of the association between BMI SDS and IGF-1 SDS in this population? and (2) Do TSH, FT3, FT4, and the FT3/FT4 ratio exert significant mediating effects in this association? The age range of 2–9 years was deliberately selected for the following reasons: children in this age interval are essentially prepubertal, thereby minimizing the confounding effects of pubertal sex steroids on the GH-IGF-1 axis and the thyroid axis; and the lower limit of 2 years avoids the physiologically high and highly variable IGF-1 levels of infancy and early toddlerhood, allowing a clearer identification of nutrition-related regulatory signals. We hypothesized that in this population, BMI and IGF-1 exhibit a linear positive association, and that peripheral conversion efficiency (FT3/FT4 ratio) serves as a mediator independent of the central TSH pathway. Given the bidirectional relationship between thyroid hormones and IGF-1 described above, we additionally conducted a reverse mediation analysis as an exploratory sensitivity assessment, in which IGF-1 served as the independent variable and BMI as the outcome, to evaluate whether thyroid axis hormones also transmit signals in the opposite direction.

## 2 Subjects and methods

### 2.1 Study design and participants

This was a retrospective cross-sectional study. We consecutively enrolled children aged 2.0–9.0 years who attended the Child Health Care Department at Wuhan Children’s Hospital due to growth concerns (e.g., short stature, decelerated annual growth velocity, or failure to thrive) between February 2022 and November 2024. This study was approved by the Ethics Committee of Wuhan Children’s Hospital (Approval No. 2026R051-E01). Informed consent was waived due to the retrospective and anonymized nature of the data.

### 2.2 Inclusion criteria

Participants were included if they met all the following criteria: (1) aged 2.0–9.0 years; (2) presented with short stature/growth retardation; (3) height SDS < -1.0, encompassing both clinical short stature (SDS < -2.0) and mild growth retardation (-2.0 ≤ SDS < -1.0); (4) prepubertal status (girls < 8.0 years, boys < 9.0 years); and (5) complete data on age, sex, height, weight, IGF-1, TSH, FT3, and FT4.

### 2.3 Exclusion criteria

Individuals were excluded for any of the following: (1) confirmed Growth Hormone Deficiency (GHD) and currently receiving recombinant human growth hormone (rhGH) therapy; (2) diagnosed thyroid disorders and receiving relevant medication; (3) diabetes mellitus; (4) central precocious puberty receiving Gonadotropin-Releasing Hormone agonist (GnRHa) therapy; (5) chronic inflammatory diseases, hepatopathy, nephropathy, or cyanotic congenital heart disease; (6) genetic syndromes (e.g., Turner, Prader-Willi, or Noonan syndromes); (7) major surgery, trauma, or infectious hospitalization within the preceding 3 months; (8) TSH > 10 μIU/mL with FT4 below the lower normal limit (clinical hypothyroidism), or TSH below the lower normal limit with markedly elevated FT3/FT4 (hyperthyroidism); and (9) verified recording errors for height, weight, or BMI SDS values outside the ± 5 range.

### 2.4 Data collection and quality control

Data on age, sex, height (cm), weight (kg), IGF-1 (ng/mL), TSH (μIU/mL), FT3 (pmol/L), and FT4 (pmol/L) were extracted from the electronic medical record system. For data cleaning, records with BMI SDS values outside the -5 to +5 range were considered potential data entry errors and were verified against the original medical records or excluded. For individuals with BMI SDS < -3, original height and weight measurements were individually reviewed and confirmed before inclusion.

### 2.5 Definitions and standardization

Anthropometric measurements: recumbent length/height and weight were measured by two trained nurses. Age- and sex-specific standard deviation scores for height (Height SDS), weight (Weight SDS), and body mass index (BMI SDS) were calculated based on the World Health Organization (WHO) Child Growth Standards.

Serum TSH, FT4, and FT3 were measured using chemiluminescence immunoassays. The institutional normal reference ranges for children were: TSH, 0.51–6.27 mIU/L; FT3, 5.20–8.60 pmol/L; and FT4, 12–22 pmol/L. The FT3/FT4 ratio was calculated as FT3 (pmol/L)/FT4 (pmol/L).

IGF-1 SDS was calculated using the LMS method based on the Chinese pediatric reference values established by the PRINCE study (14), with IGF-1 measured via chemiluminescence immunoassay.

### 2.6 Prepubertal status determination and handling of GH data

#### 2.6.1 Determination of prepubertal status and sensitivity validation

Given the retrospective design lacking systematic Tanner staging records and gonadotropin assay data, the primary analysis employed internationally accepted conservative age cutoffs (girls < 8.0 years, boys < 9.0 years) as a proxy for prepubertal status. The absence of Tanner staging and gonadotropin measurements in the electronic medical records was primarily because the vast majority of children in the enrolled age range exhibited no clinical signs of pubertal development, and therefore such assessments were not routinely performed or recorded in this clinical setting. To validate the robustness of this strategy, a predefined sensitivity analysis was conducted with stricter age cutoffs (girls < 7.0 years, boys < 8.0 years). At these stricter thresholds, the probability of true central precocious puberty is negligible, representing an unequivocally prepubertal subgroup. Consistency between the primary and this stricter subgroup analysis would indicate that using age-based criteria did not introduce significant bias in lieu of Tanner staging.

#### 2.6.2 Growth hormone data availability and handling of undiagnosed GHD

Although GH provocative testing is a key diagnostic tool for GHD, it was not systematically performed in this retrospective cohort because many children presented with mild short stature or growth deceleration without overt clinical suspicion of severe GHD. Therefore, normal GH status could not be directly confirmed for all participants. We excluded children with an established diagnosis of GHD who were receiving recombinant human GH therapy. To further reduce potential confounding from undiagnosed severe GH deficiency, we conducted a prespecified sensitivity analysis excluding children with IGF-1 SDS < -2.0, and we performed subgroup analyses according to severity of short stature (height SDS ≤ -2.0 vs. -2.0 to -1.0). The core results remained consistent across these analyses.

### 2.7 Statistical analysis

All analyses were performed using R software (version 4.4.0). After Shapiro-Wilk normality testing, continuous variables were presented as mean ± standard deviation for normally distributed data, or as median (interquartile range) for skewed data. Categorical variables were expressed as frequencies (percentages).

Primary Analysis 1: Shape of the BMI–IGF-1 Association. Using IGF-1 SDS as the dependent variable and BMI SDS as the independent variable via a restricted cubic spline (RCS) with 3 knots (at the 10th, 50th, and 90th percentiles), a RCS regression model was constructed adjusting for sex, chronological age and height SDS. The P-value for overall association and the P-value for non-linearity were reported. If the Wald test for the non-linear component yielded P > 0.05, the data were considered more supportive of a linear relationship. Based on this, the change in IGF-1 SDS per 1-unit increment in BMI SDS (β) and its 95% confidence interval (CI) were further estimated.

Primary Analysis 2: Mediating Effects of Thyroid Axis Hormones. Bootstrap resampling (5,000 replications) was employed for mediation analysis to test the indirect effects of TSH, FT3, FT4, and the FT3/FT4 ratio in the association between BMI SDS and IGF-1 SDS. All mediation models were adjusted for sex, chronological age and height SDS. Based on Hayes’ PROCESS macro (Model 4), four distinct mediation pathways were constructed: (1) BMI SDS → TSH → IGF-1 SDS; (2) BMI SDS → FT3 → IGF-1 SDS; (3) BMI SDS → FT4 → IGF-1 SDS; and (4) BMI SDS → FT3/FT4 ratio → IGF-1 SDS. For each pathway, the total effect (c path), direct effect (c’ path), indirect effect (a × b path), and the mediation proportion (indirect effect/total effect × 100%) with bias-corrected 95% CIs were reported. A bias-corrected nonparametric percentile method was used to estimate the 95% CI for the indirect effect; if this CI did not contain zero, the mediating effect was deemed statistically significant (equivalent to P < 0.05).

Sensitivity Analyses: To validate the robustness of the primary findings, three predefined sensitivity analyses were conducted: (1) stricter prepubertal definition, restricting the age threshold to girls < 7.0 years and boys < 8.0 years, with all mediation models re-run; (2) exclusion of overweight/obese children (BMI SDS > 2.0) to eliminate potential confounding effects of obesity on the thyroid axis; and (3) exclusion of children with suspected GHD (IGF-1 SDS < -2.0) to test consistency in a non-GHD subsample.

Subgroup Analyses: To explore potential heterogeneity, subgroup mediation analyses were performed by sex (boys vs. girls), age group (≤5.14 years vs. >5.14 years, based on the median split), and severity of growth retardation (height SDS < -2.0 vs. -2.0 to -1.0). In each subgroup, the mediation models were fitted using the same four thyroid axis mediators. To avoid over-adjustment, the stratifying variable was not included as a covariate within its corresponding subgroup model; other covariates (age, sex, and height SDS as appropriate) were retained.

Interaction Tests: To formally assess whether sex, age group, or severity of growth retardation moderated the BMI–thyroid axis paths, interaction terms were tested in the full cohort. Specifically, we constructed models with BMI SDS × sex, BMI SDS × age group, and BMI SDS × height SDS interactions for the a paths (BMI SDS → each thyroid axis indicator). If a significant interaction was detected (P < 0.05), the corresponding subgroup mediation results were interpreted as reflecting genuine moderation rather than random variation.

Reverse Mediation Analysis: To evaluate the possibility of reverse causation, an exploratory reverse mediation analysis was performed with IGF-1 SDS as the independent variable, BMI SDS as the dependent variable, and TSH, FT3, FT4, and the FT3/FT4 ratio as mediators. Bootstrap resampling (5,000 replications) was used to estimate indirect effects, adjusting for age, sex, and height SDS.

*Post-hoc* Statistical Power Analysis: For the four indirect effect pathways, *post-hoc* statistical power was estimated using Monte Carlo simulation based on the observed effect sizes. Specifically, 1,000 bootstrap resamples were drawn from the original dataset with replacement; within each resample, the indirect effect was estimated using 500 bootstrap replications. The proportion of simulations in which the indirect effect was statistically significant (bias-corrected 95% CI excluding zero) was taken as the estimated power. Power > 0.80 was considered indicative of adequate statistical power.

E-value Sensitivity Analysis: For significant indirect effects, E-values were calculated to quantify the minimum strength of unmeasured confounding required to shift the observed indirect effect to null. E-values were computed based on the lower confidence limit of the indirect effect.

A two-sided significance level of α = 0.05 was established for all hypothesis tests.

## 3 Results

### 3.1 Participant enrollment and baseline characteristics

A total of 3,342 children met the initial age screening criteria during the study period. After rigorous application of the inclusion and exclusion criteria, 1,242 children were included in the primary analysis (**Figure 1**).

**Figure 1:**
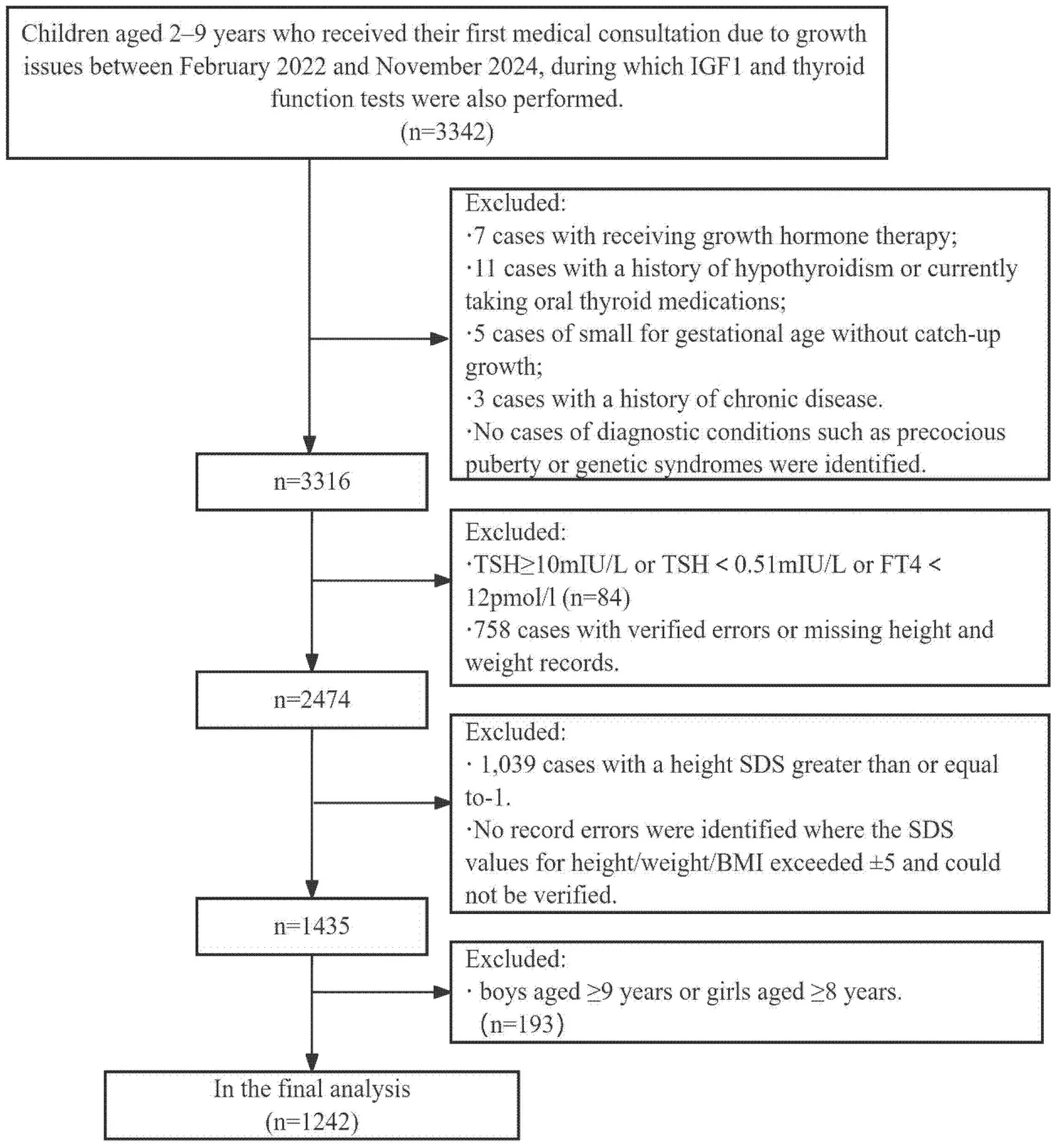
Flowchart of participants involved in the analysis.

Baseline characteristics are detailed in **Table 1**. The cohort was characterized by pronounced short stature and leanness: mean age 5.30 ± 1.53 years; 786 boys (63.29%) and 456 girls (36.71%); mean height SDS -1.57 ± 0.46; mean BMI SDS -0.45 ± 0.92; and mean IGF-1 SDS -1.35 ± 0.75. Notably, only 41 children (3.3%) had an IGF-1 SDS ≥ 0, with the remainder exhibiting negative values.

**Table 1:** Baseline characteristics of the study population, stratified by height SDS < -2.0 or between -2.0 and -1.0.

Variables	Total (n = 1242)	-2 ≤ Height SDS < -1 (n = 1045)	Height SDS < -2 (n = 197)	Statistic	P value
Demographic					
Age (years), Mean ± SD	5.30 ± 1.53	5.34 ± 1.55	5.06 ± 1.42	t = 2.39	0.017
Sex, n (%)				χ² = 0.14	0.708
Boys	786 (63.29)	659 (63.06)	127 (64.47)		
Girls	456 (36.71)	386 (36.94)	70 (35.53)		
Anthropometric					
Height SDS, Mean ± SD	-1.57 ± 0.46	-1.42 ± 0.27	-2.38 ± 0.37	t = 42.52	< 0.001
Weight SDS, Mean ± SD	-1.30 ± 0.69	-1.20 ± 0.63	-1.83 ± 0.71	t = 12.46	< 0.001
BMI SDS, Mean ± SD	-0.45 ± 0.92	-0.46 ± 0.91	-0.37 ± 0.97	t = -1.24	0.214
Thyroid function					
TSH (mIU/L), Mean ± SD	3.15 ± 1.52	3.13 ± 1.51	3.30 ± 1.56	t = -1.47	0.143
FT3 (pmol/L), Mean ± SD	6.42 ± 0.74	6.42 ± 0.73	6.42 ± 0.78	t = 0.02	0.986
FT4 (pmol/L), Mean ± SD	16.47 ± 1.80	16.46 ± 1.78	16.48 ± 1.89	t = -0.14	0.888
FT3/FT4 ratio, Mean ± SD	0.39 ± 0.05	0.39 ± 0.05	0.39 ± 0.05	t = 0.18	0.858
Growth factor					
IGF-1 SDS, Mean ± SD	-1.35 ± 0.75	-1.31 ± 0.74	-1.53 ± 0.77	t = 3.65	< 0.001

All continuous variables are presented as mean ± SD; categorical variables are presented as n (%). TSH, thyroid-stimulating hormone; FT4, free thyroxine; FT3, free triiodothyronine; IGF-1, insulin-like growth factor-1; SDS, standard deviation score. Two-tailed independent-samples t-tests were performed for group comparisons. No multiple-comparison correction was applied.

Spearman correlation analysis showed that BMI SDS was positively correlated with IGF-1 SDS (r = 0.159, P < 0.001), FT3 (r = 0.081, P < 0.01), and the FT3/FT4 ratio (r = 0.115, P < 0.001), but was not significantly correlated with TSH (P > 0.05) or FT4 (r = −0.048, P = 0.088). The FT3/FT4 ratio was positively correlated with IGF-1 SDS (r = 0.152, P < 0.001). These pairwise correlations provided preliminary support for the subsequent mediation analyses.

### 3.2 Linear association between BMI SDS and IGF-1 SDS

RCS regression analysis (**Figure 2**) demonstrated that, after adjustment for sex, age and height SDS, the overall association between BMI SDS and IGF-1 SDS was highly significant (P-overall < 0.001), whereas the non-linear component was not significant (P-nonlinear = 0.0719), supporting a purely linear model. Linear regression indicated that for every 1-unit increment in BMI SDS, IGF-1 SDS increased by 0.1148 on average (95% CI: 0.0709 to 0.1586, P < 0.001).

**Figure 2:**
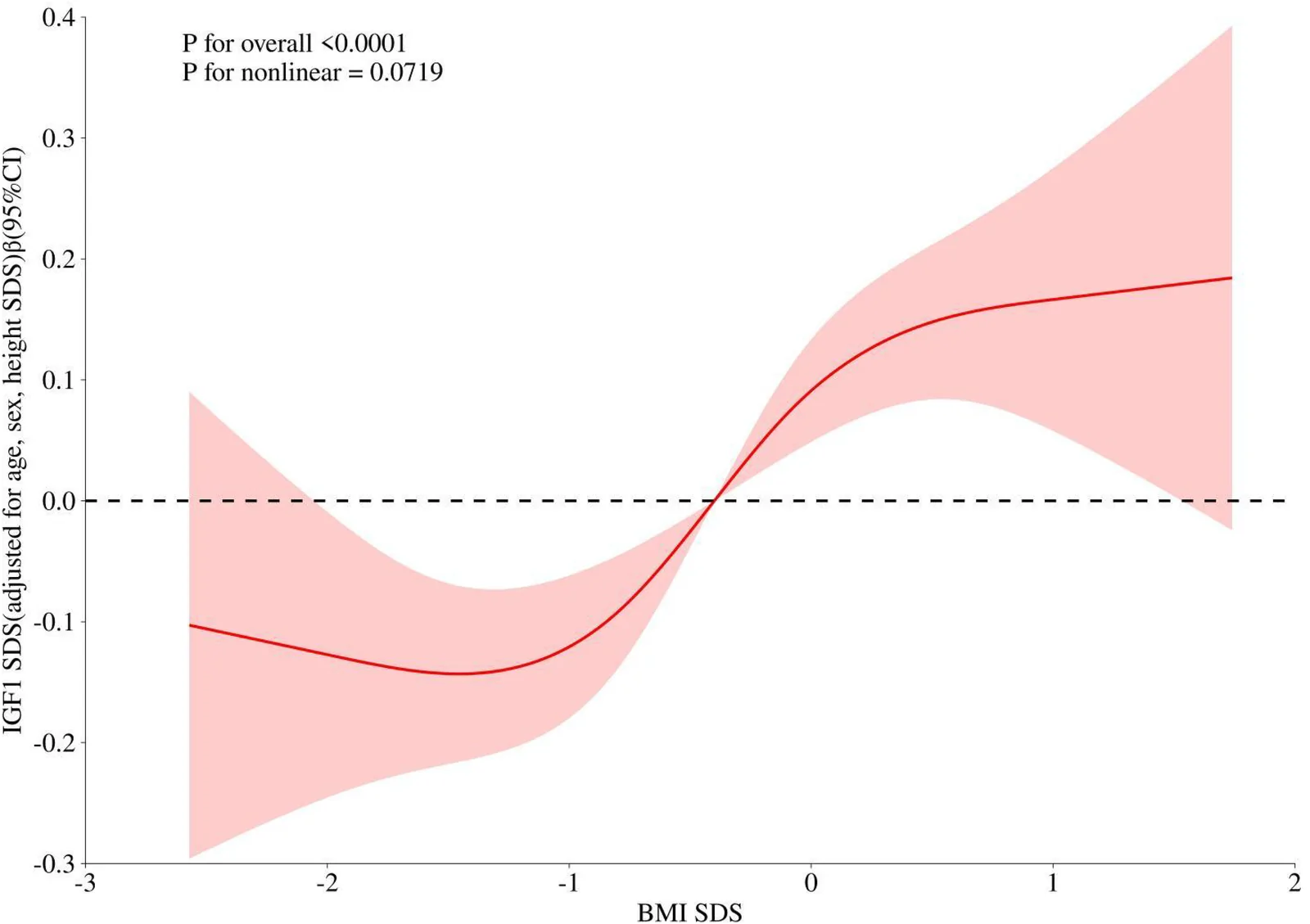
Restricted cubic spline (RCS) curve illustrating the dose-response association between BMI SDS and IGF-1 SDS (n = 1242). All analyses were adjusted for sex, chronological age and height SDS. The solid red line shows the fitted smooth curve; the shaded pink area represents the 95% confidence interval of the fitted values. The horizontal dashed line at IGF-1 SDS = 0 indicates the reference baseline. Note that the 95% confidence intervals overlap with the baseline at the lower and upper extremes of BMI SDS, which is largely attributable to reduced sample size and wider standard error at the tails of BMI SDS distribution. The P-value for the overall association was < 0.0001, whereas the P-value for nonlinearity was 0.0719, suggesting no statistically significant nonlinear relationship.

### 3.3 Mediating effects of thyroid axis hormones

Bootstrap mediation analysis (5,000 resamples) independently tested the indirect effects of TSH, FT3, FT4, and the FT3/FT4 ratio in the BMI SDS-IGF-1 SDS association. All models were adjusted for age and sex. Results are summarized in **Table 2** and **Figure 3**.

**Table 2:** Bootstrap mediation analysis results summary.

Mediator	Effect	Estimate (b)	95% bootstrap CI	*p*-value
FT3/FT4 ratio	Indirect (ACME)	0.0105	0.0042, 0.0184	< 0.001***
	Direct (ADE)	0.1043	0.0615, 0.1474	< 0.001***
	Total	0.1148	0.0723, 0.1579	< 0.001***
	Prop. Mediated	0.0912	0.0363, 0.1778	< 0.001***
FT3	Indirect (ACME)	0.0133	0.0027, 0.0243	0.0108*
	Direct (ADE)	0.1015	0.0591, 0.1433	< 0.001***
	Total	0.1148	0.0724, 0.1573	< 0.001***
	Prop. Mediated	0.1160	0.0254, 0.2342	0.0108*
FT4	Indirect (ACME)	-0.0045	-0.0108, 0.0002	0.0624
	Direct (ADE)	0.1193	0.0765, 0.1623	< 0.001***
	Total	0.1148	0.0719, 0.1577	< 0.001***
	Prop. Mediated	-0.0392	-0.1042, 0.0018	0.0624
TSH	Indirect (ACME)	0.0026	-0.0009, 0.0078	0.1504
	Direct (ADE)	0.1121	0.0699, 0.1542	< 0.001***
	Total	0.1148	0.0726, 0.1571	< 0.001***
	Prop. Mediated	0.0230	-0.0086, 0.0746	0.1504

*N* = 1242. Bootstrap resamples = 5000 for confidence-interval estimation (percentile method). Estimates are unstandardized regression coefficients (*b*). ACME, average causal mediation effect(Indirect effect); ADE, average direct effect; Prop. Mediated, Proportion Mediated; CI, confidence interval. Mediation models were adjusted for sex, chronological age and height SDS. Significance levels: *p < 0.05, **p < 0.01, ***p < 0.001.

**Figure 3:**
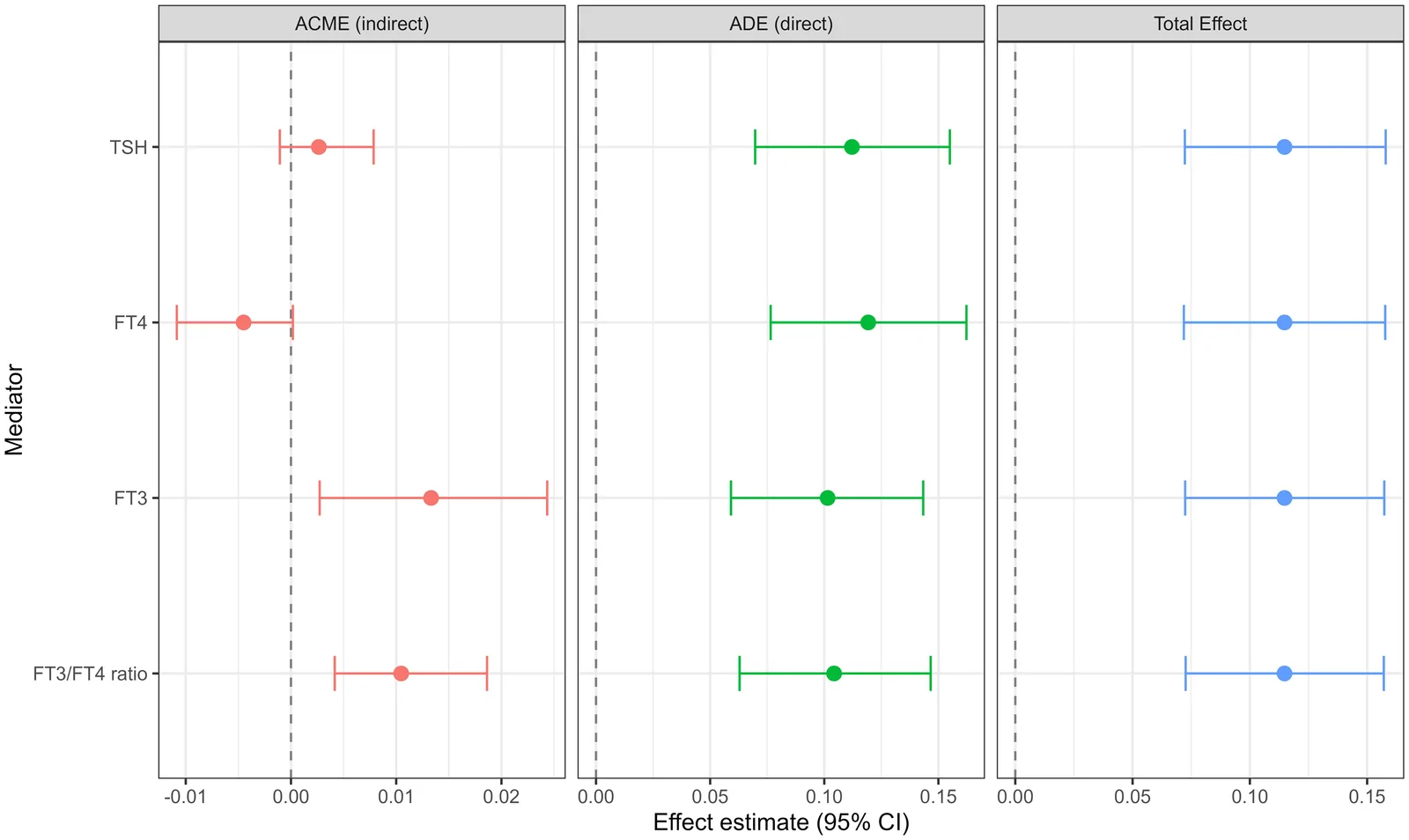
Forest plot of mediation effects for thyroid-related biomarkers (TSH, FT4, FT3/FT4 ratio, FT3) as mediators (n = 1242). Columns represent ACME (average causal mediation effect, indirect effect, red), ADE (average direct effect, green), and total effect (blue), respectively. Each dot denotes the point estimate of the effect, and horizontal bars indicate the 95% confidence intervals derived from 5000 bootstrap resamples (percentile method). The vertical dashed line at zero marks the null effect threshold; confidence intervals that do not cross zero indicate statistically significant effects. Significant mediation pathways involving FT3, FT3/FT4 ratio, TSH and FT4 are highlighted with dashed lines. Mediation models were adjusted for sex, chronological age and height SDS.

- FT3/FT4 ratio pathway: The indirect effect was significant (b = 0.0105, 95% Bootstrap CI: 0.0042 to 0.0184, P < 0.001), demonstrating the strongest statistical significance among all pathways, with a mediation proportion of approximately 9.12%.
- FT3 pathway: The indirect effect was significant (b = 0.0133, 95% Bootstrap CI: 0.0027 to 0.0243, P = 0.0108), with a mediation proportion of approximately 11.60%.
- FT4 pathway: The indirect effect reached borderline statistical significance (P = 0.0624), and the 95% CI for the indirect effect contained zero, indicating non-significance.
- TSH pathway: The path from BMI SDS to TSH was not significant (P > 0.05), and the 95% CI for the indirect effect contained zero, indicating no mediation.

### 3.4 Sensitivity analyses

Sensitivity analysis results are detailed in **Supplementary Table S1**. (1) Restricted age cutoffs: After restricting the analysis to the unequivocally prepubertal subgroup (girls < 7.0 years, boys < 8.0 years), the mediating effects of the FT3/FT4 ratio and FT3 remained robustly significant (both P < 0.05). The indirect effects of TSH and FT4 showed borderline significance (P = 0.0844 and P = 0.0872, respectively), not reaching the prespecified statistical threshold. (2) Exclusion of overweight/obese children: After excluding children with BMI SDS > 2.0, the mediating effects of the FT3/FT4 ratio and FT3 remained significant (both P < 0.05), while those of TSH and FT4 remained non-significant. (3) Exclusion of suspected GHD children: After excluding children with IGF-1 SDS < -2.0, the mediating effect of FT3 was no longer significant, whereas the mediating effect of the FT3/FT4 ratio remained significant (P = 0.0208), with an effect direction entirely consistent with the primary analysis. This contrast suggests that the mediating effect of FT3 may predominantly reflect compensatory activation of the peripheral thyroid axis under severe GH deficiency, whereas the FT3/FT4 ratio-mediated conversion efficiency regulation operates independently of GHD severity, representing a more universal fine-tuning mechanism in the nutrition-growth axis.

Collectively, these results indicate that the mediating effect of the FT3/FT4 ratio maintained statistical significance across all conditions, establishing it as the most robust transmission pathway within the thyroid axis.

### 3.5 *Post-hoc* statistical power and E-value

Monte Carlo simulation-based *post-hoc* power analysis (**Table 3**) showed that the estimated power for the indirect effect of the FT3/FT4 ratio was 91.66%, for FT3 was 67.41%, for FT4 was 28.42%, and for TSH was 10.59%. This indicates that the FT3/FT4 ratio mediation result was adequately powered, whereas the null findings for TSH and FT4 may partly reflect insufficient statistical power.

**Table 3:** *Post-hoc* Monte-Carlo statistical power and E-value assessment for causal mediation models.

Mediator	Indirect effect, % (prop. mediated)	P-value	Monte Carlo power	E-value (CI lower)
FT3/FT4 Ratio	9.12%	< 0.001***	0.9166	1.0764
FT3	11.60%	0.0108*	0.6741	1.0609
FT4	–	0.0624	0.2842	1.0000
TSH	–	0.1504	0.1059	1.0000

N = 1242. Monte-Carlo *post-hoc* power was calculated for each indirect effect. E-value (lower bound) represents the minimum strength of an unmeasured confounder that could alter the statistical significance of the observed indirect effect. Prop. Mediated (%) denotes the proportion of the total effect mediated by each biomarker. Prop. Mediated, proportion mediated; E-value, E-value for unmeasured confounding sensitivity. Significance levels: *p < 0.05, **p < 0.01, ***p < 0.001.

E-value sensitivity analysis showed that the E-values (based on the lower confidence limit) for the FT3/FT4 ratio and FT3 indirect effects were 1.0764 and 1.0609, respectively, suggesting that a relatively weak unmeasured confounder could shift the observed indirect effects to null. Therefore, the mediation findings should be interpreted as exploratory and require prospective validation.

### 3.6 Subgroup analyses

Subgroup mediation analyses were performed by sex, age group, and severity of growth retardation.The FT3/FT4 ratio remained a significant mediator in all subgroups, with consistent positivedirection (**Supplementary Table S2**).

Sex-stratified analyses. The FT3/FT4 ratio showed significant mediation in both girls and boys (proportion mediated: 16.06% and 6.63%, respectively). FT3 mediation was significant only in girls (23.2%) and not in boys.

Age-stratified analyses (≤ 5.14 years vs. > 5.14 years, based on the median). The FT3/FT4 ratio remained significant in both the ≤ 5.14 years group and the > 5.14 years group (13.67% and 9.84%, respectively). FT3 mediation was significant only in the older group (13.53%).

Severity-stratified analyses. In the severe short stature subgroup (height SDS < -2.0; n = 197), only the FT3/FT4 ratio exhibited a significant indirect effect (proportion mediated: 19.39%, P < 0.05), whereas FT3 and TSH did not. In the milder subgroup (height SDS -2.0 to -1.0; n = 1045), the FT3/FT4 ratio, FT3, and TSH all showed significant indirect effects (proportion mediated: 7.59%, 10.12%, and 3.06%, respectively; all P < 0.05). The BMI SDS × height SDS interaction was significant for the BMI → TSH path (P = 0.0022), formally supporting that the severity of growth retardation moderates this pathway.

Interaction tests (**Supplementary Table S3**) for sex and age on the BMI → FT3 and BMI → FT3/FT4 ratio paths were not significant (all P > 0.05), suggesting that the subgroup differences in FT3 mediation may reflect cumulative effect variation rather than statistically robust single-dimension moderation.

### 3.7 Reverse mediation analysis

In the reverse mediation model with IGF-1 SDS as the independent variable and BMI SDS as theoutcome (**Supplementary Table S4**), the indirect effects through FT4 and the FT3/FT4 ratio were significant (proportion mediated: −4.79% and 8.44%, respectively; P = 0.0148 and P < 0.001). However, the total indirect effect in the reverse model (approximately 3.7%) was substantially smaller than that in the forward model (approximately 21%). These findings suggest the presence of bidirectional regulatory signals, but the forward model predominated in effect size, supporting the forward nutrition → thyroid → IGF-1 pathway as the primary direction of transmission.

## 4 Discussion

Using a strictly screened cohort of prepubertal children with growth retardation (height SDS < -1.0), this study applied Bootstrap mediation analysis to elucidate the transmission characteristics of the thyroid axis in the BMI–IGF-1 association. To our knowledge, this is the first study to formally test and confirm the mediating role of the FT3/FT4 ratio in the BMI–IGF-1 association in children within a formal mediation framework. Our principal findings can be summarized in three tiers: (1) a robust linear positive association between BMI and IGF-1 was observed in this population, with no detectable inverted U-shaped inflection point; (2) the central TSH pathway was not involved in mediating this association; and (3) peripheral FT3/FT4 ratio and FT3 each exhibited significant but moderate partial mediating effects, accounting for 9.49% and 11.81% of the total effect, respectively, with the FT3/FT4 ratio demonstrating the strongest statistical significance (P < 0.001) and representing the only thyroid axis metric that passed all sensitivity analyses. Importantly, all mediation results reported herein reflect statistical associations derived from cross-sectional data and should not be interpreted as proven causal effects.

### 4.1 Clinical implications of the linear association

In contrast to the previously reported inverted U-shaped relationship between BMI and IGF-1 in a large general pediatric population (n = 3,227, 2–18 years) (6), our study observed only a linear positive component in children with growth retardation, with a non-significant non-linear component (P = 0.0719). The physiological explanation for this discrepancy is straightforward: our cohort had a BMI distribution concentrated at the lower end (mean BMI SDS negative), not yet reaching the severity and duration of obesity required to induce hepatic GH resistance (15–17); moreover, the prepubertal stage ensures relative quiescence of sex steroid modulation on the growth axis (18, 19), further excluding confounding by obesity-induced inhibitory signals. Critically, this linear finding has direct clinical translational value — it unequivocally indicates to clinicians that for lean children with growth retardation, even marginal BMI improvements can translate linearly into elevated IGF-1 levels (β = 0.1148, 95% CI: 0.0709 to 0.1586, P < 0.001), providing a quantifiable physiological basis for sustained nutritional intervention benefits.

### 4.2 Peripheral conversion efficiency, not central instructions, is key

The most innovative finding of our study is the mediating role of the FT3/FT4 ratio (P < 0.001), whereas the TSH and FT4 pathways exhibited no mediating effects. This finding suggests that at the low end of the nutritional spectrum represented by growth retardation, the set point of the central hypothalamic-pituitary-thyroid axis remains relatively stable, not triggering an “energy-sparing” or “stress hyper-TSH” program in response to mild leanness. In contrast to the quiescent central pathway, the peripheral conversion pathway was significantly activated. The FT3/FT4 ratio reflects the activity of type I and type II deiodinases — the peripheral tissue capacity to convert the prohormone T4 to active T3 (11, 20). The respective indirect effects of the FT3/FT4 ratio and FT3 in the individual mediation analyses should be interpreted as a composite estimate of the peripheral thyroid pathway rather than two entirely independent routes. The FT3/FT4 ratio exhibited stronger statistical significance and clearer physiological meaning than FT3 alone because it corrects for substrate FT4 concentration, directly reflecting the functional indicator of conversion efficiency. The negative, albeit non-significant, indirect effect of FT4 (b = -0.0045) appears counterintuitive but is actually entirely consistent with the aforementioned “peripheral conversion” hypothesis. Physiologically, nutritional improvement upregulates peripheral deiodinase activity, accelerating T4-to-T3 conversion, leading to a consumptive decline in FT4. The negative direction of the FT4 effect paradoxically corroborates the sensitivity and specificity of the FT3/FT4 ratio as a conversion efficiency indicator — the ratio increases not merely by FT3 elevation, but through the combined effect of rising FT3 and declining FT4, genuinely reflecting upregulated deiodinase activity. Our findings suggest that during nutritional status improvement in growth-retarded children, the organism does not globally mobilize the thyroid axis by upregulating central commands (TSH); rather, it upregulates deiodinase activity in peripheral target tissues such as the liver to more efficiently utilize available T4 resources, locally synergizing with GH to promote IGF-1 synthesis. This reveals a “thrifty” peripheral regulatory strategy that achieves refined enhancement of growth factor synthesis at minimal cost of central disturbance.

Consistency between Descriptive Correlation and Mediation Analysis: Complementary Evidence forCentral Quiescence and Peripheral Activation. The results of descriptive correlation analyses andmediation analyses in our study were highly concordant, collectively pointing to the core conclusion of “central non-involvement, peripheral key involvement.” In the correlation matrix (**Supplementary Table S5**), BMI SDS showed no significant correlations with either TSH or FT4 (both P > 0.05), and the correlation between TSH and FT4 was also non-significant (P > 0.05). These three “non-correlations” stand in stark contrast to the classic pattern of a significant positive correlation between BMI and TSH observed in obese children. In the obese state, leptin secreted by adipose tissue stimulates hypothalamic Thyrotropin-Releasing Hormone (TRH) neurons, which in turn upregulates pituitary TSH secretion, resulting in a robust positive BMI–TSH correlation (12). However, at the low end of the nutritional spectrum — growth retardation — the degree of energy deficit has not yet reached the threshold to suppress the central thyroid axis; the set point of the hypothalamic-pituitary-thyroid axis remains relatively stable under mild leanness, neither pushed upward by leptin due to “energy excess” nor suppressed by “energy insufficiency.” In other words, the central thyroid axis exhibits “inertial stability” at the lean end, as opposed to the “sensitized responsiveness” at the obese end. The non-correlation between FT4 and TSH further suggests that in this population with BMI SDS in the lower-normal range, FT4 fluctuations are likely determined by intrinsic thyroid autoregulation or thyroid-binding globulin, rather than driven by immediate central TSH modulation.

In sharp contrast, BMI SDS was significantly positively correlated with IGF-1 SDS, the FT3/FT4 ratio, and FT3 (all P < 0.05); and the FT3/FT4 ratio, FT3, and FT4 were all significantly positively correlated with IGF-1 SDS (P < 0.001 for all). These significant bivariate associations provided the preliminary basis for subsequent mediation analyses — the correlations of BMI with the FT3/FT4 ratio and FT3 supported path a, the correlations of the FT3/FT4 ratio, FT3, and FT4 with IGF-1 supported path b, and the BMI–IGF-1 correlation confirmed the existence of the total effect.

It is particularly noteworthy that in the correlation matrix, TSH was also significantly positively correlated with IGF-1 SDS (P < 0.01), yet the indirect effect of TSH in the Bootstrap mediation model was not significant. This seemingly paradoxical phenomenon has a clear methodological implication: the bivariate TSH–IGF-1 SDS correlation may represent a confounding correlation driven by BMI as a common driver — that is, nutritional status simultaneously influences TSH (via leptin) and IGF-1 (via the GH-insulin pathway), but TSH itself is not an independent transmission pathway through which BMI affects IGF-1. This precisely underscores the necessity of employing formal mediation analysis rather than relying solely on descriptive correlations.

Furthermore, the pattern of a significant FT4–IGF-1 SDS correlation (P < 0.001) alongside a non-significant BMI SDS–FT4 correlation (P > 0.05) — a “path a absent, path b present” pattern — indirectly corroborates that nutritional status does not regulate growth factors by altering the absolute secretion of thyroid hormones (FT4, TSH), but rather through upregulating peripheral deiodinase activity and enhancing T4-to-T3 conversion efficiency (FT3/FT4 ratio). In summary, the descriptive correlations (bivariate associations) and the mediation analysis (causal transmission) collectively paint a complete regulatory picture: the central thyroid axis maintains a stable set point at the lean end, and nutritional signals bypass central commands to directly promote IGF-1 synthesis in peripheral target tissues by increasing conversion efficiency.

### 4.3 Systematic comparison with previous studies

The core innovation of our study lies in being the first to formally integrate BMI, the thyroid axis, and IGF-1 into a complete regulatory network within a mediation analysis framework, rather than merely reporting bivariate correlations. Previously, multiple large observational studies have examined the BMI–IGF-1 association in children via cross-sectional and cohort designs. A cross-sectional study of 3,227 children aged 2–18 years showed that IGF-1 initially increased and then decreased with rising BMI, with a significant decline observed in the obese subgroup (6). An Egyptian study in healthy children aged 2–8 years found lower IGF-1 in overweight/obese children but only conducted group comparisons without involving the thyroid axis (21). Surup et al., using the large LIFE Child cohort, demonstrated positive correlations of BMI with TSH and FT3, and a negative correlation with FT4, but did not connect these findings to the growth axis (12). Recently, Jiang et al. found lower IGF-1 and higher BMI in obese boys with mild subclinical hypothyroidism; however, their analysis was limited to group comparisons and did not test the BMI → TSH → IGF-1 mediation pathway (22). In summary, all previous studies have stopped at bivariate associations or group comparisons; none have integrated them into a formal mediation model. The incremental contribution of our study lies not in discovering new bivariate associations, but in being the first to quantify the transmission effect and the upper limit of contribution of the thyroid axis within this network.

Furthermore, our findings align with and extend a broader international body of work on the regulation of peripheral thyroid hormone metabolism under nutritional stress. In fasting and malnutrition, peripheral deiodinase activity is downregulated, leading to reduced T3 and a decreased FT3/FT4 ratio, a hallmark of the non-thyroidal illness syndrome (23, 24). Conversely, nutritional recovery restores deiodinase activity and normalizes the FT3/FT4 ratio. At the molecular level, type 1 and type 2 deiodinase expression and activity are tightly regulated by nutritional signals, including insulin and leptin (25, 26). In pediatric populations, population-based reference data for the FT3/FT4 ratio have been established, confirming the physiological relevance of this index (20). Our study extends these observations by demonstrating — within a formal mediation framework — that the FT3/FT4 ratio, but not central TSH, partially transmits the effect of BMI on IGF-1 in prepubertal growth-retarded children. To our knowledge, this represents the first quantitative integration of the nutrition–thyroid–growth axis into a mediation model.

### 4.4 Scientific value and clinical interpretation of the moderate effect size

It must be candidly acknowledged that the mediation proportions for the FT3/FT4 ratio and FT3 in the full population were 9.12% and 11.60%, respectively — moderate effect sizes. However, the “statistical credibility” and “biological contribution” of an effect are two independent dimensions. The mediating effect of the FT3/FT4 ratio (P < 0.001) remained robust across all sensitivity analyses, indicating that the mediation by peripheral conversion efficiency is genuinely present at the population level and not a spurious finding. In a complex, multi-pathway, multi-node regulatory network such as the nutrition-endocrine-growth axis, any single mediating variable explaining nearly one-tenth of the total effect has clear biological significance. The value of our study lies not in claiming that the thyroid axis is the primary pathway connecting nutrition and growth, but in providing the first rigorous statistical evidence that peripheral conversion efficiency is an independent and credible regulatory node in this association, while quantifying its upper contribution limit. This finding provides a reference and hypothesis basis for future efforts to construct a more comprehensive “nutrition-endocrine-growth axis” mediation network.

Additionally, E-value sensitivity analyses indicated that the E-values for the FT3/FT4 ratio and FT3 indirect effects were 1.0764 and 1.0609, respectively. These values suggest that a relatively weak unmeasured confounder could theoretically shift the observed indirect effects to null. Accordingly, the reported mediation proportions should be interpreted as upper-bound estimates, and the true causal contribution of peripheral thyroid hormone conversion efficiency may be smaller. Nevertheless, the consistency of the FT3/FT4 ratio mediation across all sensitivity and subgroup analyses provides some reassurance that this pathway is unlikely to be merely a statistical artifact.

### 4.5 Distinct pathways revealed by sensitivity analyses

Sensitivity analyses further revealed qualitative differences in regulatory robustness betweenthe FT3/FT4 ratio and FT3. After excluding children with suspected GHD (IGF-1 SDS < -2.0), the mediating effect of FT3 disappeared (P = 0.1068), whereas that of the FT3/FT4 ratio remained robust (P = 0.0208). This indicates that the significance of FT3 as a mediator is partially dependent on compensatory activation of the thyroid axis triggered by severe GH deficiency—under extreme GH insufficiency, the organism may substantially upregulate peripheral deiodinase activity and FT3 levels in an attempt to “bypass” the GH deficiency bottleneck to maintain residual IGF-1 synthesis. Conversely, the conversion efficiency regulation reflected by the FT3/FT4 ratio operates independently of GHD severity, representing a universal fine-tuning mechanism across the growth retardation spectrum. This contrast methodologically further supports the robustness of the FT3/FT4 ratio as a core mediating indicator, and physiologically reveals possible differences in thyroid axis regulatory patterns between severe GHD and mild growth retardation. Furthermore, after further restricting age to the unequivocally prepubertal subgroup (girls < 7.0 years, boys < 8.0 years) and excluding overweight/obese children (BMI SDS > 2.0), the mediating effects of the FT3/FT4 ratio and FT3 remained significant (**Supplementary Table S1**), indicating that the core conclusions are not driven by occult pubertal onset or extreme values from a minority of overweight children.

Subgroup analyses (**Supplementary Table S2**) by severity of growth retardation further revealed a graded pattern. In the severe short stature subgroup (height SDS < -2.0), only the FT3/FT4 ratio exhibited a significant mediating effect, whereas FT3 and TSH did not. In the milder subgroup (height SDS -2.0 to -1.0), the FT3/FT4 ratio, FT3, and TSH all showed significant indirect effects. The BMI SDS × height SDS interaction was significant for the BMI → TSH path (P = 0.0022), formally supporting that growth retardation severity moderates this pathway. This gradient suggests that central TSH compensation operates only in milder growth retardation, whereas severe short stature is characterized by central suppression and reliance on peripheral conversion efficiency as the sole thyroid axis mediator.

### 4.6 Clinical translational prospects

Our findings offer a novel perspective for refined assessment and stratified intervention in children with growth retardation. First, for lean children with growth retardation, proactive nutritional intervention is expected to linearly improve IGF-1 without concern for a “threshold effect” of overnutrition. Second, when evaluating thyroid axis status in children with growth retardation, clinicians should not focus solely on absolute TSH, FT3, or FT4 levels; rather, they should pay attention to changes in the FT3/FT4 ratio, which may more sensitively reflect whether nutritional intervention has genuinely “activated” the peripheral thyroid pathway. If only an increase in the FT3/FT4 ratio is observed without concurrent IGF-1 changes, other nutrition-growth pathways (e.g., insulin, Insulin-like Growth Factor Binding Protein-3) should be considered for potential impediments. Third, despite significant descriptive correlations between TSH and IGF-1 in our study, the mediation effect was non-significant. This discrepancy warns clinicians that the simple correlation between TSH and IGF-1 may be driven by BMI as a common driver and should not be directly interpreted as an independent causal regulation of TSH on IGF-1. Fourth, to provide a preliminary frame of reference for future clinical evaluation, we describe the FT3/FT4 ratio distribution observed in this cohort. The FT3/FT4 ratio showed a median of 0.39 (IQR: 0.36–0.43), with a P5–P95 range of 0.31–0.48. It must be emphasized that this is a descriptive, cohort-specific range rather than a validated clinical reference interval; it cannot be directly extrapolated to healthy children or to other clinical populations. Given the cross-sectional nature of these data, longitudinal within-individual changes in the FT3/FT4 ratio during nutritional intervention may ultimately prove more informative than any single threshold value. Standardized age- and sex-specific reference intervals should be established in future multi-center studies with healthy reference populations.

### 4.7 Strengths and limitations

The strengths of our study include the following. First, the relatively large sample size (N = 1,242) provided sufficient statistical power; stringent age restriction (2–9 years) minimized gonadal axis confounding. Second, we employed RCS for non-linearity exploration and Bootstrap mediation analyses to enhance inferential rigor. Third, multi-layered sensitivity analyses (restricted age cutoffs, exclusion of overweight/obese, exclusion of suspected GHD) verified the robustness of our findings. Fourth, we are the first to report the mediating role of the FT3/FT4 ratio in the BMI–IGF-1 association in growth-retarded children, filling a gap in the integration of nutrition-thyroid-growth axis components into a formal mediation model. Fifth, we additionally performed subgroup analyses, interaction tests, reverse mediation analysis, Monte Carlo power analysis, and E-value sensitivity analysis, providing a comprehensive robustness assessment.

Several limitations must be acknowledged. First, the cross-sectional design precludes the establishment of strict causal relationships. Our mediation model is predicated on a well-established biological temporal sequence (“altered nutritional status → adaptive thyroid axis adjustments → hepatic IGF-1 synthesis changes”), which is physiologically well-grounded (5, 27–29). However, we cannot entirely exclude reverse or bidirectional causality — IGF-1 could theoretically influence thyroid structure/function via promoting follicular epithelial proliferation (1), thereby modulating energy metabolism and BMI. We empirically evaluated this possibility through a reverse mediation analysis, in which IGF-1 SDS served as the independent variable and BMI SDS as the outcome. The total indirect effect in the reverse direction was substantially smaller than that in the forward direction (approximately 3.65% vs. 20.72%), suggesting that the forward nutrition → thyroid → IGF-1 axis predominates, with only a limited bidirectional component. Nevertheless, these findings should be interpreted as statistical associations rather than causal evidence. Future prospective studies should employ cross-lagged panel designs to formally test bidirectional temporal sequences. Second, the lack of bone age data precluded direct assessment of skeletal maturation and growth potential; however, IGF-1, as a core endocrine mediator of linear growth, has intrinsic research value in its association with BMI and thyroid axis independent of bone age. Third, several growth-related confounders — including parental height SDS, daily nutritional intake, chronic low-grade inflammatory markers, and insulin levels — were not available in this retrospective cohort. These unmeasured factors may bias the observed mediation proportions. We addressed this concern by adjusting all models for height SDS as a proxy for growth potential and by conducting E-value sensitivity analyses, which indicated that the observed indirect effects are sensitive to relatively weak unmeasured confounding. Therefore, the reported mediation proportions should be regarded as upper-bound estimates. Fourth, the single-center design may limit generalizability to other pediatric populations of different ethnicities and geographic regions; external validation in multi-center, multi-ethnic cohorts is warranted. Fifth, complete GH provocative testing data were not available for all participants. Although we excluded children with an established diagnosis of GHD on rhGH therapy and performed sensitivity analyses excluding those with IGF-1 SDS < -2.0, we cannot completely rule out the presence of undiagnosed mild GHD. Seventh, although our sex-stratified analyses showed a consistent positive direction for the FT3/FT4 ratio mediation in both sexes, the FT3 mediation was significant only in girls. However, formal interaction tests did not support significant sex moderation of the BMI → FT3 path, suggesting that this difference may reflect effect variation rather than a robust sex-specific mechanism. Dedicated future studies with larger sex-specific samples are needed to further evaluate potential gender differences in the nutrition–thyroid–growth axis. Eighth, we did not explore potential sex differences in the above associations and mediating effects in depth, which warrants dedicated future exploration.

## 5 Conclusions

In summary, in prepubertal children aged 2–9 years with growth retardation (height SDS < -1.0): (1) BMI and IGF-1 exhibit a robust linear positive association, providing a physiological basis for the expected sustained benefits of clinical nutritional intervention; (2) peripheral thyroid hormone conversion efficiency (FT3/FT4 ratio) is statistically supported as an independent and credible regulatory node linking nutritional status and growth factors, whereas the central TSH pathway is not involved — revealing a “thrifty” peripheral regulatory strategy that achieves refined enhancement of growth factor synthesis at minimal cost of central disturbance; (3) although this peripheral pathway exhibits a moderate effect size (approximately 9.12%), it is statistically highly robust (P < 0.001); its value lies in being the first to quantify the upper contribution limit of the thyroid axis in nutrition-growth regulation, offering a key reference for subsequent systematic comparisons with other pathways; and (4) the high consistency between descriptive correlations and mediation analyses — BMI SDS showing no significant correlations with TSH or FT4, while BMI SDS, FT3/FT4 ratio, FT3, and IGF-1 are all significantly intercorrelated — multidimensionally supports the core finding of “central quiescence and peripheral activation”. These findings should be interpreted as exploratory statistical associations, given the cross-sectional design; reverse mediation analysis suggested a weaker reverse direction, and E-value sensitivity analyses indicated susceptibility to unmeasured confounding. Preliminary data from this cohort suggest that monitoring the FT3/FT4 ratio may offer greater clinical informational value than measuring individual thyroid hormone levels alone, although longitudinal validation is required.

## Data Availability

The raw data supporting the conclusions of this article will be made available by the authors, without undue reservation.
