# Supplementary material for: Mediating role of peripheral thyroid hormone conversion efficiency in the association between BMI and IGF-1 in children with growth retardation: a cross-sectional study
Source: Front Endocrinol (Lausanne). 2026 Sep 17;17:1941425. doi: 10.3389/fendo.2026.1941425 (PMC13627020; doi:10.3389/fendo.2026.1941425)
Supplement: Supplementary file 1 [file Table1.docx]

Table S1. Sensitivity Analysis of Mediation Effects Under Different Exclusion Criteria

| **Mediation Pathway** | **Exclusion Criterion** | **N** | **Indirect Effect b(95% CI)** | **Prop. Mediated b(95% CI)** | **P value** |
| --- | --- | --- | --- | --- | --- |
| **BMI SDS - FT3/FT4 Ratio - IGF-1 SDS** |  |  |  |  |  |
| Main analysis | None | 1242 | 0.0105 (0.0042, 0.0184) | 0.0912 (0.0363, 0.1778) | < 0.001*** |
| Sensitivity 1 | Age-restricted(Girls<7,Boys<8) | 1123 | 0.0102 (0.0037, 0.0186) | 0.0927 (0.0335, 0.1957) | 0.0012** |
| Sensitivity 2 | Excluding BMI SDS>2.0 | 1239 | 0.0102 (0.0039, 0.0183) | 0.0896 (0.0350, 0.1807) | < 0.001*** |
| Sensitivity 3 | Excluding IGF-1 SDS<-2.0 | 1019 | 0.0039 (0.0004, 0.0090) | 0.0537 (0.0052, 0.1487) | 0.0208* |
| **BMI SDS - FT3 - IGF-1 SDS** |  |  |  |  |  |
| Main analysis | None | 1242 | 0.0133 (0.0027, 0.0243) | 0.1160 (0.0254, 0.2342) | 0.0108* |
| Sensitivity 1 | Age-restricted(Girls<7,Boys<8) | 1123 | 0.0114 (0.0003, 0.0232) | 0.1031 (0.0030, 0.2274) | 0.0448* |
| Sensitivity 2 | Excluding BMI SDS>2.0 | 1239 | 0.0129 (0.0020, 0.0244) | 0.1140 (0.0190, 0.2341) | 0.0208* |
| Sensitivity 3 | Excluding IGF-1 SDS<-2.0 | 1019 | 0.0055 (-0.0013, 0.0133) | 0.0751 (-0.0207, 0.2221) | 0.1068 |
| **BMI SDS - FT4 - IGF-1 SDS** |  |  |  |  |  |
| Main analysis | None | 1242 | -0.0045 (-0.0108, 0.0002) | -0.0392 (-0.1042, 0.0018) | 0.0624 |
| Sensitivity 1 | Age-restricted(Girls<7,Boys<8) | 1123 | -0.0037 (-0.0092,  0.0004) | -0.0335 (-0.0982, 0.0037) | 0.0844 |
| Sensitivity 2 | Excluding BMI SDS>2.0 | 1239 | -0.0043 (-0.0104, 0.0006) | -0.0384 (-0.1089, 0.0046) | 0.0768 |
| Sensitivity 3 | Excluding IGF-1 SDS<-2.0 | 1019 | -0.0031 (-0.0081, 0.0008) | -0.0417 (-0.1405, 0.0115) | 0.1316 |
| **BMI SDS - TSH - IGF-1 SDS** |  |  |  |  |  |
| Main analysis | None | 1242 | 0.0026 (-0.0009, 0.0078) | 0.0230 (-0.0086, 0.0746) | 0.1504 |
| Sensitivity 1 | Stricter prepubertal age cotoffs (Girls<7yr,Boys<8yr) | 1123 | 0.0030 (-0.0003, 0.0084) | 0.0275 (-0.0028, 0.0852) | 0.0872 |
| Sensitivity 2 | Excluding BMI SDS>2.0 | 1239 | 0.0022 (-0.0013, 0.0074) | 0.0196 (-0.0121, 0.0710) | 0.2396 |
| Sensitivity 3 | Excluding IGF-1 SDS<-2.0 | 1019 | 0.0028 (-0.0005, 0.0078) | 0.0380 (-0.0072, 0.1231) | 0.1068 |

Bootstrap resamples = 5000 for confidence‑interval estimation (percentile method). Estimates are unstandardized regression coefficients (*b*). Abbreviations: Prop. Mediated, Proportion Mediated; CI, confidence interval. Mediation models were adjusted for sex, chronological age and height SDS. Significance levels: * *p* < 0.05, ** *p* < 0.01, *** *p* < 0.001.

Table S2. Subgroup-specific mediation analyses stratified by sex, chronological age, and height SDS to assess heterogeneity of mediation effects.

| **Subgroup** | **Mediator** | **N** | **Indirect Effect b(95% CI)** | **Prop. Mediated b(95% CI)** | **P-value** |
| --- | --- | --- | --- | --- | --- |
|  | **FT3/FT4 Ratio** |  |  |  |  |
| Sex: Girls |  | 456 | 0.0172 (0.0042, 0.0347) | 0.1606(0.0360,0.5695) | 0.0056** |
| Sex: Boys |  | 786 | 0.0077 (0.0011, 0.0165) | 0.0663(0.0095,0.1613) | 0.0184* |
| Chronological age ≤ 5.14 years (median cutoff) |  | 621 | 0.0090 (0.0011, 0.0190) | 0.1367(0.0041,0.6687) | 0.0452* |
| Chronological age > 5.14 years (median cutoff) |  | 621 | 0.0145 (0.0038, 0.0292) | 0.0984(0.0266,0.2218) | 0.0032** |
| Height SDS < -2 |  | 197 | 0.0196 (0.0002, 0.0496) | 0.1939(-0.0780,1.2413) | 0.0476* |
| -2 ≤ Height SDS < -1 |  | 1045 | 0.0090 (0.0028, 0.0174) | 0.0759(0.0244,0.1660) | 0.0028** |
|  | **FT3** |  |  |  |  |
| Sex: Girls |  | 456 | 0.0248 (0.0045, 0.0471) | 0.2320(0.0398,0.7351) | 0.0152* |
| Sex: Boys |  | 786 | 0.0078 (-0.0039, 0.0210) | 0.0671 (-0.0368, 0.1934) | 0.1976 |
| Chronological age ≤ 5.14 years (median cutoff) |  | 621 | 0.0120 (-0.0013, 0.0259) | 0.1844 (-0.0719, 0.8563) | 0.0724 |
| Chronological age > 5.14 years (median cutoff) |  | 621 | 0.0199 (0.0012, 0.0399) | 0.1353 (0.0082, 0.2932) | 0.0376* |
| Height SDS < -2 |  | 197 | 0.0174 (-0.0027, 0.0417) | 0.1724 (-0.0996, 1.0295) | 0.0944 |
| -2 ≤ Height SDS < -1 |  | 1045 | 0.0120 (-0.0000, 0.0250) | 0.1012 (-0.5015, 0.2266) | 0.0496* |
|  | **FT4** |  |  |  |  |
| Sex: Girls |  | 456 | -0.0061 (-0.0189, 0.0029) | -0.0571 (-0.2738. 0.0280) | 0.2000 |
| Sex: Boys |  | 786 | -0.0038 (-0.0108, 0.0015) | -0.0325 (-0.1112, 0.0123) | 0.1652 |
| Chronological age ≤ 5.14 years (median cutoff) |  | 621 | -0.0035 (-0.0120, 0.0027) | -0.0540 (-0.4074, 0.0754) | 0.2700 |
| Chronological age > 5.14 years (median cutoff) |  | 621 | -0.0054 (-0.0158, 0.0028) | -0.0364 (-0.1188, 0.0182) | 0.1720 |
| Height SDS < -2 |  | 197 | -0.0031 (-0.0188, 0.0065) | -0.0307 (-0.3635, 0.1584) | 0.5840 |
| -2 ≤ Height SDS < -1 |  | 1045 | -0.0043 (-0.0111, 0.0013) | -0.0364 (-0.1064, 0.0104) | 0.1188 |
|  | **TSH** |  |  |  |  |
| Sex: Girls |  | 456 | 0.0005 (-0.0072, 0.0100) | 0.0050 (-0.1017, 0.1080) | 0.9064 |
| Sex: Boys |  | 786 | 0.0036 (-0.0007, 0.0111) | 0.0311 (-0.0060, 0.1044) | 0.1276 |
| Chronological age ≤ 5.14 years (median cutoff) |  | 621 | 0.0055 (-0.0007, 0.0152) | 0.0855 (-0.0225, 0.5474) | 0.1020 |
| Chronological age > 5.14 years (median cutoff) |  | 621 | 0.0005 (-0.0039, 0.0062) | 0.0034 (-0.0290, 0.0426) | 0.8220 |
| Height SDS < -2 |  | 197 | -0.0073 (-0.0297, 0.0073) | -0.0729 (-0.6057, 0.1388) | 0.3588 |
| -2 ≤ Height SDS < -1 |  | 1045 | 0.0036 (0.0000, 0.0098) | 0.0306 (0.0001, 0.0916) | 0.0488* |

Bootstrap resamples = 5000 for confidence‑interval estimation (percentile method). Estimates are unstandardized regression coefficients (b). Abbreviations: CI, confidence interval; Prop. Mediated, proportion mediated. Each subgroup mediation model was adjusted for the remaining covariates (e.g., chronological age and height‑SDS for sex‑stratified analyses). The chronological‑age subgroup cutoff was defined according to the sample median of 5.14 years. Note that the proportion‑mediated metric should be interpreted with caution when the indirect effect is close to zero. Significance levels: * *p* < 0.05, ** *p* < 0.01, *** *p* < 0.001.

Table S3. Interaction tests for sex, chronological age, and height SDS on path a (X → M) and path b (M → Y) of mediation models.

| **Interaction** | **Mediator** | **Path a (X → M)** | **Path b (M → Y)** |
| --- | --- | --- | --- |
| BMI SDS * Sex | FT3/FT4 Ratio | 0.1685 | 0.8988 |
|  | FT3 | 0.1579 | 0.7592 |
|  | FT4 | 0.7842 | 0.6502 |
|  | TSH | 0.4281 | 0.7358 |
| BMI SDS * Chronological Age | FT3/FT4 Ratio | 0.9830 | 0.6744 |
|  | FT3 | 0.7330 | 0.1037 |
|  | FT4 | 0.6538 | 0.1176 |
|  | TSH | 0.4310 | 0.9072 |
| BMI SDS * Height SDS | FT3/FT4 Ratio | 0.6534 | 0.6744 |
|  | FT3 | 0.3545 | 0.1747 |
|  | FT4 | 0.1426 | 0.6106 |
|  | TSH | 0.0022* | 0.5822 |

*N* = 1242. Path a represents the coefficient for the exposure‑to‑mediator pathway (X → M); path b represents the coefficient for the mediator‑to‑outcome pathway (M → Y). Interaction terms include BMI SDS × sex, BMI SDS × chronological age and BMI SDS × height SDS,. All models were adjusted for sex, chronological age, and height SDS. Significance levels: * *p* < 0.05, ** *p* < 0.01, *** *p* < 0.001.

Table S4. Reverse causal mediation analyses treating IGF-1 SDS as exposure, thyroid biomarkers as mediators, and BMI SDS as outcome, with E-value for unmeasured confounding.

| **Mediation Pathway** | **Indirect Effect, Estimate(95%CI)** | **Prop. Mediated,Estimate(95%CI)** | **P-value** | **E-value(CI lower)** |
| --- | --- | --- | --- | --- |
| IGF1 SDS - FT3/FT4 ratio - BMI SDS | 0.0153 (0.0054, 0.0276) | 0.0844 (0.0290, 0.1714) | < 0.001*** | 1.0792 |
| IGF1 SDS - FT3 - BMI SDS | 0.0115 (-0.0066, 0.0295) | 0.0633 (-0.0355, 0.1917) | 0.2052 | 1.0000 |
| IGF1 SDS - FT4 - BMI SDS | -0.0087 (-0.0185, -0.0014) | -0.0479 (-0.1144, -0.0076) | 0.0148* | 1.0000 |
| IGF1 SDS - TSH - BMI SDS | 0.0031 (-0.0030, 0.0110) | 0.0169 (-0.0179, 0.0664) | 0.3128 | 1.0000 |

*N* = 1242. Reverse mediation models regarded IGF‑1 SDS as the exposure, FT3/FT4 ratio, FT3, FT4, and TSH as mediators, and BMI‑SDS as the outcome. Bootstrap resamples = 5000 for confidence‑interval estimation (percentile method). Estimates are unstandardized regression coefficients. Abbreviations: CI, confidence interval; Prop. Mediated, proportion mediated; E‑value (lower), lower bound of E‑value for unmeasured confounding. All reverse mediation models were adjusted for sex, chronological age, and height‑SDS. Significance levels: * *p* < 0.05, ** *p* < 0.01, *** *p* < 0.001.

Table S5. Pearson correlation matrix for the main variables (n = 1242).

| **Variable** | **BMI SDS** | **TSH** | **FT3** | **FT4** | **FT3/FT4 Ratio** | **IGF-1 SDS** |
| --- | --- | --- | --- | --- | --- | --- |
| **BMI SDS** | 1 |  |  |  |  |  |
| **TSH** | 0.045 | 1 |  |  |  |  |
| **FT3** | 0.081** | 0.067* | 1 |  |  |  |
| **FT4** | -0.048 | -0.022 | 0.330*** | 1 |  |  |
| **FT3/FT4 Ratio** | 0.115*** | 0.073* | 0.604*** | -0.544*** | 1 |  |
| **IGF-1 SDS** | 0.159*** | 0.087** | 0.255*** | 0.096*** | 0.152*** | 1 |

Correlation coefficient magnitude: *r* < 0.2 indicates weak correlation; 0.2 ≤ *r* < 0.4 indicates moderate correlation; *r* ≥ 0.4 indicates strong correlation. Note: The FT3/FT4 ratio is a derived variable calculated as FT3 divided by FT4. Strong correlations between the ratio and its constituent hormones (FT3, FT4) may partly reflect mathematical construction rather than purely biological associations. Significance levels: * *p* < 0.05, ** *p* < 0.01, *** *p* < 0.001.
